# Supplementary material for: Risk of a seizure recurrence after a breakthrough seizure and the implications for driving: further analysis of the standard versus new antiepileptic drugs (SANAD) randomised controlled trial
Source: BMJ Open. 2017 Jul 10;7(7):e015868. doi: 10.1136/bmjopen-2017-015868 (PMC5726069; doi:10.1136/bmjopen-2017-015868)
Supplement: Author's manuscript [file bmjopen-2017-015868.draft_revisions.pdf]

BMJ Open

Risk of a seizure recurrence after a breakthrough seizure and the implications for driving - further analysis of the Standard versus New Antiepileptic Drugs (SANAD) Randomised Controlled Trial

Journal:	*BMJ Open*
Manuscript ID	bmjopen-2017-015868
Article Type:	Research
Date Submitted by the Author:	05-Jan-2017
Complete List of Authors:	Bonnett, L; University of Liverpool, Biostatistics<br>Powell, Graham; University of Liverpool, Molecular and Clinical Pharmacology<br>Tudur-Smith, Catrin; University of Liverpool, Biostatistics<br>Marson, Anthony; University of Liverpool, Department of Molecular and Clinical Pharmacology
**Primary Subject Heading**:	Neurology
Secondary Subject Heading:	Evidence based practice
Keywords:	Epilepsy < NEUROLOGY, Adult neurology < NEUROLOGY, STATISTICS & RESEARCH METHODS

SCHOLARONE™
Manuscripts

Dear Editor,

We would be grateful if you would consider our paper for publication in BMJ Open.

The UK based Driving and Vehicle Licensing Agency (DVLA) guidance suggests that people who have had an unprovoked, breakthrough, seizure after a period of remission whilst on treatment are usually allowed to regain their group one, ordinary, driving licence one year after the seizure recurrence provided they have been seizure free. This is based on an assumption that their risk of a seizure in the next 12 months is below 20%. However, this is extremely difficult for clinicians to judge in the absence of relevant evidence.

There are no published data that tabulate seizure recurrence risks conditional upon having been seizure free for specific periods of time following a breakthrough seizure. There is therefore a dearth of data to inform driving policy and advice given to patients in the UK or elsewhere.

Our paper reports an analysis of data from the study of Standard and New Antiepileptic Drugs (SANAD) and provides data that can inform driving policy. In particular we identify the characteristics of patients at higher risk who may pose a risk to themselves and the general public should they return to driving. In addition to supporting DVLA guidance regarding time off driving, the data also raise important questions around risk stratification and how estimates and confidence intervals should be used to inform evidence based policy.

Given that this is a reanalysis of a previously published randomised controlled trial we have not provided a CONSORT statement. However, figure 1 of the manuscript is equivalent to a CONSORT flow diagram.

Yours sincerely

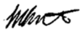

L J Bonnett

NIHR Post-Doctoral Fellow

For peer review only

For peer review only - http://bmjopen.bmj.com/site/about/guidelines.xhtml

RISK OF A SEIZURE RECURRENCE AFTER A BREAKTHROUGH SEIZURE AND THE IMPLICATIONS FOR DRIVING - FURTHER ANALYSIS OF THE STANDARD VERSUS NEW ANTIEPILEPTIC DRUGS (SANAD) RANDOMISED CONTROLLED TRIAL

LJ BONNETT, GA POWELL, C TUDUR SMITH, AG MARSON

Corresponding Author
Laura J. Bonnett, NIHR Post-Doctoral Fellow
Department of Biostatistics,
Waterhouse Building, Block F,
1-5 Brownlow Street,
Liverpool,
L69 3GL
Email: L.J.Bonnett@liverpool.ac.uk
Telephone: 0151 795 9686

Co-authors
Graham A Powell, MRC Clinical Training Fellow, Department of Molecular and Clinical Pharmacology, Clinical Sciences Centre, Lower Lane, Fazakerley, Liverpool, L9 7LJ. U.K.

Catrin Tudur Smith, Reader in Biostatistics, Department of Biostatistics, Waterhouse Building, Block F, 1-5 Brownlow Street, University of Liverpool, Liverpool, L69 3GL, U.K.

Anthony G Marson, Professor of Neurology, Department of Molecular and Clinical Pharmacology, Clinical Sciences Centre, Lower Lane, Fazakerley, Liverpool, L9 7LJ. U.K.

Key words: Epilepsy, Adults, Driving, Breakthrough Seizures

Word count: 3334

Number of tables: 4

Number of figures: 2

Number of references: 29

ABSTRACT

Objectives: A breakthrough seizure is one occurring after at least 12 months seizure freedom whilst on treatment. The Driver and Vehicle Licensing Agency (DVLA) allow an individual to return to driving once they have been seizure free for 12 months following a breakthrough seizure. This is based on the assumption that the risk of a further seizure in the next 12 months has dropped below 20%. This analysis considers whether the prescribed one year off driving following a breakthrough seizure is sufficient for this, and stratifies risk according to clinical characteristics.

Design, Setting, Participants, Interventions & Main outcome measures: The multi-centre United Kingdom based Standard Versus New Antiepileptic Drug (SANAD) Study was a randomised controlled trial assessing standard and new antiepileptic drugs for patients with newly diagnosed epilepsy. For participants aged at least 16 with a breakthrough seizure, data have been analysed to estimate the annual seizure recurrence risk following a period of six, nine and 12 months seizure freedom. Regression modelling was used to investigate how antiepileptic drug treatment and a number of clinical factors influence the risk of seizure recurrence.

Results: At 12 months following a breakthrough seizure the overall unadjusted risk of a recurrence over the next 12 months is lower than 20%, risk 17% (95% confidence interval: 15% to 19%). However, some patient subgroups have been identified which have an annual recurrence risk significantly greater than 20% after an initial 12 month seizure free period following a breakthrough seizure.

For peer review only - http://bmjopen.bmj.com/site/about/guidelines.xhtml

Conclusions: This reanalysis of SANAD provides estimates of seizure recurrence risks following a breakthrough seizure that will inform policy and guidance about regaining an ordinary driving license. Further guidance is needed as to how such data should be utilised.

Trial Registration: SANAD is registered with the International Standard Randomized Controlled Trial Number Register - ISRCTN38354748.

ARTICLE SUMMARY

Strengths and limitations of this study

- This reanalysis of SANAD provides estimates of seizure recurrence risks following a breakthrough seizure that will inform policy and guidance about regaining an ordinary driving license.
- The SANAD data largely reflects patients with newly diagnosed epilepsy so we have been unable to explore longer term patterns of seizures.
- Patients with epilepsy may elect not to report breakthrough seizures to their clinicians or the relevant driving authority which may lead to an under-estimation of risk.

For peer review only

For peer review only - http://bmjopen.bmj.com/site/about/guidelines.xhtml

INTRODUCTION

A breakthrough seizure is defined as the first seizure after a minimum of 12 months seizure freedom whilst on treatment. The legislation(1) that directs the decisions of the United Kingdom Driver and Vehicle Licensing Agency (DVLA) is informed by a risk based approach. This is summarised in guidance available on their website.(2) In The Motor Vehicle Regulation, epilepsy is defined as a history of two or more clinically unprovoked seizures.(1) According to this, people who have had a breakthrough seizure are usually allowed to regain their group one (ordinary) driving licence one year after the breakthrough seizure provided they have been seizure free, based on the assumption that their risk of a seizure in the next 12 months has fallen below 20%.

There are currently few published studies in which seizure recurrence risks are estimated and factors that modify risk investigated. Existing publications(3-6) have focussed on recurrence immediately following a first seizure, or recurrence after treatment withdrawal. Only Bonnett 2010(5) and Bonnett 2011(6) have presented risks of recurrence in the next 12 months following seizure freedom at time points such as six or 12 months. There are no publications considering risk of recurrence following breakthrough seizures. There is therefore a need for reliable published data to inform decisions made by clinicians, DVLA guidance and/or European Union legislation, and legislation outside the European Union.

The SANAD trial compared standard and new antiepileptic drugs as monotherapy. Arm A recruited 1721 patients (89% focal epilepsy) who were randomised to treatment with carbamazepine, gabapentin, lamotrigine, topiramate, or oxcarbazepine. Arm B recruited 716 patients (66% generalised, 27% unclassified) who were randomised to lamotrigine, topiramate, or valproate. Patients were followed up to the end of the study whether they remained on their randomised treatment or not, according to the intention to treat principle. Outcomes assessed included time to 12 month remission, time to treatment failure, and time to first seizure.

Here, data from a subset of participants achieving 12 month remission whilst on treatment followed by a breakthrough seizure have been analysed to estimate the subsequent risk of seizure recurrence. Modelling has been used to investigate how a number of clinical factors influence the outcome.

METHODS

Patients

The methods for the SANAD study have been published elsewhere.(7, 8) In summary, patients were eligible for inclusion into SANAD if, in the previous year, they had a history of at least two clinically definite unprovoked epileptic seizures and they were at least five years old. Patients were recruited into Arm A if the recruiting clinician considered carbamazepine to be the optimal standard treatment option. Between December 1st 1999 and June 1st 2001 patients were allocated in a ratio of 1:1:1:1 to carbamazepine, gabapentin, lamotrigine, and topiramate. From 1st June 2001 to 31st August 2004 an oxcarbazepine group was added to the trial and patients were randomly allocated in a ratio of 1:1:1:1:1 to carbamazepine, gabapentin, lamotrigine, oxcarbazepine or topiramate.

Patients were eligible for inclusion in Arm B if the recruiting clinician regarded valproate the standard treatment option. Participants were randomly allocated in a 1:1:1 ratio to valproate, lamotrigine or topiramate between January 12th 1999 and August 31st 2004. The two primary outcomes in SANAD were time to treatment failure from randomisation and time to the first period of 12 months of remission from seizures following randomisation.

In this paper the Arm A and Arm B datasets have been combined in order to undertake prognostic modelling stratifying by arm. In the original publications trial arms were analysed and reported separately, as the primary purpose was to compare the effectiveness of new antiepileptic drugs with the standard treatments. Here the purpose is different, the aim being to assess the risk of a seizure recurrence following a breakthrough seizure, irrespective of the specific drug that the patient was on at randomisation, or the subsequent choice of treatment.

In order to make the analysis reported here relevant to those of driving age, only participants who achieved 12 month remission whilst on treatment and then had a breakthrough seizure, and were aged 16 years or over when

For peer review only - http://bmjopen.bmj.com/site/about/guidelines.xhtml

the breakthrough seizures occurred were included. Sixteen years of age was chosen as the lower cut off as by the age 17, after 12 months of follow-up, they would be eligible for a provisional group one license in the United Kingdom. Other European Union countries have a minimum driving age of 18 years(9) with some exceptions such as Hungary(10) and Southern Ireland(11) where the limit is 17 years. In addition, the analysis only included patients who, in the six months prior to their breakthrough seizure, underwent an increase in dosage, or had no change in dosage. In other words, patients with any decrease in dose either with an intention to withdraw, or not, were excluded, as their seizure was likely to be due to antiepileptic drug withdrawal, which is handled differently in the legislation and analyses informing legislation following antiepileptic drug withdrawal have been published.(6)

Statistical Analysis

The outcome of interest is the probability of a seizure recurrence in the next 12 months having been seizure free from the breakthrough seizure to the time point in question. For example, the probability of someone who was seizure free for six months after his or her breakthrough seizure, having a seizure in months seven to 18 was calculated by dividing the probability of having a seizure by 18 months by the probability of having a seizure by six months. Risks of recurrence in the next 12 months for other time points were calculated similarly using the Cox model. Confidence intervals for estimates were calculated utilising a revised version of Greenwood's formula.(12-14) Although SANAD was a randomised trial, in this analysis the outcome was measured from the date of the breakthrough seizure, not the date of randomisation.

Our list of potential prognostic factors included: gender, febrile seizure history, first degree relative with epilepsy, neurological insult, seizure type, epilepsy type, electroencephalogram (EEG) result, computerised tomography (CT) or magnetic resonance imaging (MRI) result, total number of tonic-clonic seizures recorded prior to breakthrough seizure, age at breakthrough seizure, number of treatments required to achieve 12 month remission prior to breakthrough seizure (either monotherapy or polytherapy), time to achieve 12 month remission prior to breakthrough seizure, and breakthrough seizure treatment decision (no change to treatment plan, increase dosage, or decrease dosage for any reason). The breakthrough seizure treatment decision is defined to have occurred up to three months after the seizure and is used as a proxy for the decision that was made at the time of first clinic visit following the breakthrough seizure.

Patients were classified as having neurological insult if they had learning disabilities or neurological deficit, while EEG was classified as normal, not clinically indicated, non-specific abnormality or epileptiform abnormality (focal or generalized spikes or spike and slow wave activity). Seizure types were classified according to the International League Against Epilepsy seizure classification.(15) Epilepsy type was first classified as focal, generalised, or unclassified with the unclassified category being used when there was uncertainty between focal onset and generalised onset seizures.

Continuous variables were investigated using log and fractional polynomial transformations.(16-19) The results for the continuous variables are presented as post-hoc defined categorical variables with categories chosen according to knot positions for a spline model fit to the data.(20) Schoenfeld residual plots(21) and incorporation of time-dependent covariate effects were used to investigate the proportional hazards assumption.

Variables associated with a higher risk of seizure recurrence were determined univariably and after adjusting for multiple variables using log-rank tests and Cox proportional hazards modelling methods. A best fitting, parsimonious, multivariable model was produced with variable reduction by Akaike's Information Criterion.(22) The recurrence risk in the next 12 months for combinations of risk factors was calculated from the multivariable model.(23) All analyses were undertaken using R 3.2.3.

RESULTS

Figure 1 illustrates patient disposition of the 2627 patients recruited into both Arm A and Arm B of SANAD, and identifies patients relevant to this analysis. Table 1 summarises the patient demographics for the 399 patients under analysis. Of these patients, 254 experienced at least one further seizure after breakthrough. Patients in Arm A were followed up for a median of 1.67 years following a breakthrough seizure (interquartile

For peer review only - http://bmjopen.bmj.com/site/about/guidelines.xhtml

range (IQR) 0.85 to 2.59 years) while patients in Arm B were followed up for a median of 1.41 years (IQR 0.55 to 2.56 years). In total there were 705.6 patient years of follow-up after the breakthrough seizure.

Figure 2 illustrates the risk of seizure recurrence after a breakthrough seizure. The median time to a further seizure following a breakthrough was 76 days (IQR 57 to 122 days). The probability of a seizure by 12 months was 70.1%. Table 2 shows unadjusted 12 month seizure recurrence risks at various time points after the breakthrough seizure. At six months the estimate is significantly above 20%. At 12 months however, the estimate is below 20% and significantly so as the 95% confidence interval does not include 20%.

Table 1: Patient demographics

Characteristic (n (%) unless otherwise stated)	Arm A (n=286)	Arm B (n=113)	Total (n=399)
Male	159 (56)	72 (64)	231 (58)
Febrile seizure history	15 (5)	5 (4)	20 (5)
Epilepsy in first degree relative	24 (8)	21 (19)	45 (11)
Neurological insult	38 (13)	9 (8)	47 (12)
Seizures			
Simple or complex partial with secondary generalised seizures	180 (63)	5 (4)	185 (46)
Simple or complex partial only	72 (25)	1 (0)	73 (18)
Generalised tonic-clonic seizures only	4 (1)	32 (29)	36 (9)
Absence seizures	1 (0)	5 (5)	6 (2)
Myoclonic or absence seizures with tonic- clonic seizures	0 (0)	28 (25)	28 (7)
Tonic-clonic seizures, uncertain if focal or generalised	27 (10)	34 (30)	61 (15)
Other	2 (1)	8 (7)	10 (3)
Epilepsy type			
Partial	253 (88)	6 (5)	259 (65)
Generalised	6 (2)	69 (61)	75 (19)
Unclassified	27 (10)	38 (34)	65 (16)
EEG results			
Normal	134 (47)	32 (28)	166 (42)
Non-specific abnormality	49 (17)	13 (12)	62 (16)
Epileptiform abnormality	69 (24)	64 (57)	133 (33)
Not clinically indicated	34 (12)	4 (3)	38 (9)
CT/MRI scan results			
Normal	164 (57)	59 (52)	223 (56)
Abnormal	75 (26)	10 (9)	85 (21)
Not clinically indicated	47 (17)	44 (39)	91 (23)
Number of treatments required to achieve 12 month remission			
Monotherapy	219 (77)	86 (77)	305 (77)
Polytherapy	67 (23)	27 (23)	94 (23)
Number of tonic-clonic seizures reported by first breakthrough seizure, median (IQR)	3 (1, 6)	3 (2, 6)	3 (1, 6)
Age at first breakthrough seizure, median (IQR)	44.5 (31.8, 57.7)	24.0 (21.1, 34.5)	38.3 (24.5, 53.5)
Time to achieve 12 month remission prior to breakthrough seizure (years), median (IQR)	1.2 (1.0, 1.9)	1.1 (1.0, 1.8)	1.2 (1.0, 1.9)
Treatment decision prior to breakthrough seizure			
No change to treatment plan	261 (91)	101 (89)	362 (91)
Increase dosage	25 (9)	12 (11)	37 (9)
Breakthrough seizure treatment decision			
No change to treatment plan	169 (61)	67 (61)	236 (61)
Increase dosage	99 (36)	40 (37)	139 (36)
Decrease dosage for any reason, or missing decision	9 (3)	2 (2)	11 (3)

Table 2: Unadjusted 12 month seizure recurrence risks at time points after breakthrough seizure: risk (%, 95% Confidence Interval)

Time seizure free after breakthrough seizure (months)	Number at Risk	Risk of seizure in following 12 months
6	119	32 (28 to 36)
9	99	24 (21 to 27)
12	80	17 (15 to 19)

For peer review only - http://bmjopen.bmj.com/site/about/guidelines.xhtml

Results for univariable and multivariable modelling of time to seizure recurrence are presented in Table 3. In the univariable model, number of drugs required to achieve initial 12 month remission and time to achieve a first 12 month remission prior to breakthrough seizure were associated with seizure recurrence risk – patients requiring polytherapy to achieve remission were more likely to have a recurrence than those requiring monotherapy. Additionally, patients achieving remission immediately at one year were less likely to have a recurrence following a breakthrough seizure than those who took longer to achieve 12 month remission. Breakthrough seizure treatment decision was also associated with the outcome; patients having an increase in dose after their breakthrough seizure were more likely to have a recurrence than those not changing their treatment, which may be counter intuitive, but indicates clinicians are able to identify those at higher recurrence risk.

The final multivariable model included number of drugs required to achieve initial remission, time to achieve initial 12 month remission and breakthrough seizure treatment decision. As before – patients requiring polytherapy to achieve remission were more likely to have a recurrence than those requiring monotherapy, patients achieving remission immediately at one year were less likely to have a recurrence than those who took longer to achieve 12 month remission, and patients increasing their dose after their breakthrough seizure were more likely to have a recurrence than those not changing their dose. There was no evidence to suggest that the proportional hazards assumption, underlying the Cox model, was invalid.

Breakthrough seizure treatment decision, although significantly associated with the outcome, should not be considered as a modifiable variable, as clinicians will find it very difficult to use this information to inform treatment decisions for future patients. Therefore the model was refitted excluding this covariate, and the resulting parsimonious model included number of drugs attempted to achieve initial 12 month remission, and time taken to achieve initial 12 month remission. The direction of the effects remained unchanged.

The risk of recurrence at 12 months for patients with particular characteristics was estimated from the parsimonious multivariable regression model. Results can be seen in Table 4. At six months seizure freedom following a breakthrough seizure no patient subgroups had a risk of recurrence that was below 20%. By 12 months of seizure freedom, the current recommended time off driving following a breakthrough seizure, several patient subgroups still had estimates in excess of the 20%. In particular, the length of time required for the estimate of seizure recurrence to fall below 20% for patients requiring polytherapy to achieve initial 12 month remission, and taking three or more years to enter initial period of 12 month remission is 15 months.

For peer review only - http://bmjopen.bmj.com/site/about/guidelines.xhtml

Table 3: Effect estimates from univariable and multivariable models
[TC = tonic-clonic; Gen = generalised]

Variable	Comparison	Univariable p-value	Univariable HR (95% CI)	Multivariable HR (95% CI)	Multivariable HR (95% CI) w/o decision variable
Gender	Female<br>Male	0.43	1.00<br>1.11 (0.86, 1.42)	N/A	N/A
Febrile seizure history	Absent<br>Present	0.28	1.00<br>0.69 (0.35, 1.34)	N/A	N/A
Epilepsy in 1st degree relative	Absent<br>Present	0.82	1.00<br>1.05 (0.69, 1.59)	N/A	N/A
Neurological insult	Absent<br>Present	0.59	1.00<br>0.90 (0.62, 1.32)	N/A	N/A
Seizure type	Simple/complex partial + 2° gen.<br>Simple/complex partial only<br>Generalised TC only<br>Absence<br>Myoclonic/absence + TC<br>TC (uncertain if focal or gen.)<br>Other	 0.35<br>0.65<br>0.96<br>1.00<br>0.49<br>0.89	1.00<br>1.17 (0.84, 1.63)<br>0.87 (0.48, 1.58)<br>1.03 (0.35, 3.03)<br>1.00 (0.49, 2.03)<br>0.85 (0.54, 1.34)<br>1.07 (0.44, 2.55)	N/A	N/A
Epilepsy type	Partial<br>Generalised<br>Unclassified	 0.65<br>0.55	1.00<br>0.88 (0.52, 1.49)<br>0.88 (0.57, 1.35)	N/A	N/A
EEG results	Normal<br>Non-specific Abnormality<br>Epileptiform Abnormality<br>Not done/Missing	 0.62<br>0.87<br>0.05	1.00<br>0.91 (0.63, 1.32)<br>0.98 (0.73, 1.30)<br>0.60 (0.36, 1.00)	N/A	N/A
CT/MRI scan results	Normal<br>Abnormal<br>Not done/Missing	 0.15<br>0.86	1.00<br>0.79 (0.57, 1.09)<br>0.97 (0.71, 1.33)	N/A	N/A
No. drugs attempted for remission	Monotherapy<br>Polytherapy	0.01	1.00<br>1.47 (1.11, 1.94)	1.00<br>1.37 (1.02, 1.84)	1.00<br>1.28 (0.96, 1.71)
Number of tonic-clonic seizures reported by first breakthrough seizure	0<br>1<br>2<br>3-4<br>5-6<br>7-10<br>11-20<br>>20	0.60	1.00<br>1.00 (1.00, 1.00)<br>1.00 (1.00, 1.01)<br>1.00 (0.99, 1.01)<br>1.00 (0.99, 1.02)<br>1.01 (0.98, 1.03)<br>1.01 (0.97, 1.05)<br>1.31 (0.48, 3.52)	N/A	N/A
Age at first breakthrough seizure	≤ 20<br>21-30<br>31-45<br>46-70<br>> 70	0.39	1.00<br>1.02 (0.97, 1.07)<br>1.07 (0.92, 1.23)<br>1.14 (0.85, 1.53)<br>1.22 (0.78, 1.89)	N/A	N/A
Time to achieve initial 12 month remission (years)	1<br>1-1.5<br>1.5-2<br>2-3<br>>3	<0.001	1.00<br>1.27 (1.12, 1.44)<br>1.57 (1.24, 1.98)<br>1.75 (1.31, 2.34)<br>1.89 (1.36, 2.62)	1.00<br>1.21 (1.06, 1.38)<br>1.43 (1.12, 1.82)<br>1.56 (1.15, 2.11)<br>1.65 (1.17, 2.43)	1.00<br>1.24 (1.08, 1.41)<br>1.49 (1.16, 1.89)<br>1.64 (1.21, 2.22)<br>1.75 (1.24, 2.46)
Breakthrough seizure decision	No change to treatment plan<br>Increase dosage<br>Decrease dosage (or not specified)	 <0.001<br>0.83	1.00<br>2.05 (1.59, 2.66)<br>1.07 (0.59, 1.93)	1.00<br>2.05 (1.59, 2.66)<br>0.99 (0.55, 1.79)	N/A

HR>1 – seizure recurrence more likely

For peer review only - http://bmjopen.bmj.com/site/about/guidelines.xhtml

Table 4: Risk of seizure recurrence in next 12 months estimated from multivariable model at specific seizure-free periods.

No. drugs required to achieve remission prior to breakthrough seizure	Time to achieve 12 month remission (years) prior to breakthrough seizure	Months of seizure freedom from breakthrough seizure	Risk of seizure in next 12 months (%, 95% CI)	Months of seizure freedom required from breakthrough seizure until annual risk falls <20%
Monotherapy	1	6<br>9<br>12<br>18	20 (10 to 31)<br>15 (4 to 25)<br>10 (0 to 21)<br>6 (0 to 16)	6.1
Monotherapy	2	6<br>9<br>12<br>18	30 (21 to 39)<br>22 (13 to 32)<br>16 (6 to 26)<br>10 (0 to 19)	10.6
Monotherapy	3	6<br>9<br>12<br>18	32 (23 to 41)<br>24 (15 to 33)<br>17 (8 to 27)<br>11 (1 to 20)	11.1
Monotherapy	4	6<br>9<br>12<br>18	33 (24 to 42)<br>25 (16 to 34)<br>18 (8 to 27)<br>11 (1 to 20)	11.1
Polytherapy	2	6<br>9<br>12<br>18	37 (29 to 45)<br>28 (19 to 30)<br>20 (11 to 30)<br>12 (3 to 22)	13.2
Polytherapy	3	6<br>9<br>12<br>18	40 (32 to 48)<br>30 (22 to 39)<br>22 (13 to 31)<br>13 (4 to 23)	15.0
Polytherapy	4	6<br>9<br>12<br>18	41 (33 to 48)<br>31 (22 to 39)<br>22 (13 to 31)<br>14 (5 to 23)	15.8

For peer review only

DISCUSSION

In the United Kingdom the DVLA prescribe one year off driving following a breakthrough seizure based on legislation and the assumption that a person's risk of a seizure in the next 12 months is below 20%. According to data from the SANAD study, the overall risk of a seizure recurrence, unadjusted for any covariates, falls significantly below 20% by 12 months of seizure freedom following the breakthrough seizure as required. Covariates significantly associated with the outcome were time taken to achieve an initial 12 month remission, number of drugs required to achieve that remission, and breakthrough seizure treatment decision. As expected, those patients who achieve a period of 12 month remission quickly, and those patients who require only one drug to achieve remission, had a lower chance of a seizure recurrence.

The decision to not change antiepileptic drug dose following a breakthrough seizure was associated with a lower risk of a recurrence than the decision to increase dosage. This result is potentially counter-intuitive as one might expect an increase in dose to reduce seizure risk. However, it is likely that clinicians are able to identify patients at higher risk of recurrence and recommend treatment changes to reduce that risk, although additional relevant clinical factors have not been identified by our model, and this requires further investigation. It is important to highlight that in most cases, the decision to increase dose was taken in between neurology clinic appointments at which follow-up data were collected, presumably at the advice of the GP or neurologist. As a result, accurate dates of dose increase have not been recorded and it is possible that a subgroup of patients had further seizures following the initial breakthrough seizure, prompting the clinician to increase the antiepileptic drug dose. When breakthrough seizure treatment decision was removed from the list of candidate variables to reflect the fact that clinicians will find it very difficult to use this information to inform treatment decisions for future patients, the parsimonious model included covariates for number of drugs required to achieve an initial 12 month remission,

For peer review only - http://bmjopen.bmj.com/site/about/guidelines.xhtml

and time taken to achieve initial 12 month remission. Only patients requiring polytherapy to achieve initial 12 month remission and taking at least two years to achieve initial 12 month remission required longer than 12 months for their risk of a subsequent seizure to be less than 20%. This suggests that the current 12 month time off driving is generally appropriate. Even in the high risk groups, the recurrence risks are fairly close to 20% if the focus is on point estimates.

Few publications have considered risk of a breakthrough seizure and tend to be focused on patients in developing countries.(24, 25) A study of 256 patients in Uganda identified non-compliance to antiepileptic drug therapy, duration of treatment, infections, and menses among female study participants as factors significantly associated with breakthrough seizures.(25) Precipitating factors for breakthrough seizures for a study of 90 patients in Egypt were missed doses, sleep deprivation and psychological stress, although the authors also found differences in duration of seizure control, number of antiepileptic drugs and abnormal epileptic activity in EEG between patients with and without breakthrough seizures.(24) These factors were not collected as part of the SANAD study and as such have not been considered as part of this analysis. Neither study considered outcomes following the breakthrough study. We are unaware of any studies looking at outcome after a breakthrough seizure. In particular, we have been unable to identify any prognostic models considering risk of seizure recurrence following a breakthrough seizure for patients of driving age in developed countries. Another analysis of SANAD for patients of driving age has considered risk of a second treatment failure after a first.(26)

Limitations

SANAD recruited a large number of patients and followed them up for a long period – up to six years in some cases. However, only a small subset of these patients was relevant to address the question of risk of a seizure recurrence following a breakthrough seizure for patients of driving age. The requirement of patients to achieve initial remission of at least 12 months and then have a breakthrough seizure to be included in this analysis also meant that the follow-up of patients after the breakthrough seizure was relatively short. This means that some confidence intervals associated with the risk estimates are quite wide. Additionally, the SANAD data largely reflects patients with newly diagnosed epilepsy. We have therefore been unable to explore longer term patterns. For example, if patients go into and out of remission then their seizure recurrence risks might change compared to these estimates. The subset of patients considered for this analysis may also have limited power to detect some prognostic effects as significant. Other important factors may exist which have not been analysed, or collected.

The multivariable model for risk of seizure recurrence included a continuous covariate – time to achieve initial 12 month remission. Therefore, to estimate the risk of recurrence over the next 12 months for combinations of risk factors including this covariate, the variable had to be categorised which may not be the most efficient approach.(27) Also, neurological insult, seizure type, epilepsy type, and CT/MRI scan result were recorded at baseline rather than at the breakthrough seizure. Although these covariates may have changed by a breakthrough seizure, it is likely that any change occurred in only a small number of patients. EEG was also only recorded at baseline, and it is possible that EEG on treatment would be prognostic, although given the unpredictable nature of breakthrough seizures, it would not be feasible to undertake an EEG in order to inform risk.

There is evidence to suggest that patients with epilepsy may elect not to report breakthrough seizures to their clinicians or the relevant driving authority.(28) The evidence collected as part of SANAD is patient reported seizure counts and therefore our results may be under-estimating the actual risk. Increased patient counselling regarding the risks involved with driving, the need for driving regulations, and the importance of compliance with these rules may only have a limited impact as the implications for patients losing their driving license are potentially serious such as job losses, and resulting lack of independence. The model developed here should ideally be validated in other similar datasets. However, no other similar datasets exist. The best match is a set of individual participant data we have collected.(29) These data include only very small numbers of relevant patients. Therefore, alternative data sources are required.

Conclusions

Twelve months appears to be an appropriate time off driving for patients of driving age who have experienced a period of at least 12 months initial seizure freedom followed by a breakthrough seizure. Provided that patients

For peer review only - http://bmjopen.bmj.com/site/about/guidelines.xhtml

remain seizure free for 12 months following a breakthrough seizure, their risk of a seizure in the next 12 months would be less than the 20% risk standard that informs the UK legislation and DLVA guidance.

As discussed in depth in Bonnett 2010,(5) the legislators and DVLA need to decide whether to base time off driving on unadjusted estimates only, or whether they should consider estimates adjusted for important clinical factors. Although our unadjusted results suggest that 12 months off driving is sufficient time off driving, risk estimates differ substantially among groups. For some patient subgroups at least 15 months off driving is required for their point estimate to reduce below 20%. Additionally, discussions are required to determine whether associated 95% confidence intervals should be used to inform the decision making process. The unadjusted risk estimate is significantly below 20% by 12 months. However, none of the adjusted risk estimates are significantly below 20% by 12 months.

Implementing a policy based on clinical factors is potentially challenging. In fact, in practice time to achieve remission may be the only factor that could be incorporated into such an assessment as there is potential for manipulation of drugs in terms of number and doses to meet driving objectives. Furthermore, introducing a tiered system may compromise patient care as patients would be inclined to 'fit in' to the shorter duration if driving is important to them.

REFERENCES

1. The Motor Vehicles (Driving Licences) (Amendment) Regulations 2013, (2013).
2. Driver and Vehicle Licensing Agency. Assessing fitness to drive - a guide for medical professionals. 2016.
3. Kim LG, Johnson TL, Marson AG, Chadwick DW. Prediction of Risk of Seizure Recurrence after a Single Seizure and Early Epilepsy: Further Results from the MESS Trial. The Lancet Neurology. 2006;5(4):317-22.
4. Prognosis of Epilepsy in Newly Referred Patients: A Multicenter Prospective Study of the Effects of Monotherapy on the Long-Term Course of Epilepsy. Collaborative Group for the Study of Epilepsy. Epilepsia. 1992;33(1):45-51.
5. Bonnett LJ, Tudur-Smith C, Williamson PR, Marson AG. Risk of recurrence after a first seizure and implications for driving: further analysis of the Multicentre study of early Epilepsy and Single Seizures. BMJ. 2010;341:c6477.
6. Bonnett LJ, Shukralla A, Tudur-Smith C, Williamson PR, Marson AG. Seizure recurrence after antiepileptic drug withdrawal and the implications for driving: further results from the MRC Antiepileptic Drug Withdrawal Study and a systematic review. Journal of Neurology Neurosurgery and Psychiatry. 2011;82(12):1328-33.
7. Marson AG, Al-Kharusi AM, Alwaidh M, Appleton R, Baker GA, Chadwick DW, et al. The SANAD study of effectiveness of carbamazepine, gabapentin, lamotrigine, oxcarbazepine, or topiramate for treatment of partial epilepsy: an unblinded randomised controlled trial. Lancet. 2007;369(9566):1000-15.
8. Marson AG, Al-Kharusi AM, Alwaidh M, Appleton R, Baker GA, Chadwick DW, et al. The SANAD study of effectiveness of valproate, lamotrigine, or topiramate for generalised and unclassifiable epilepsy: an unblinded randomised controlled trial. Lancet. 2007;369(9566):1016-26.
9. Directive 2006/126/EC of the European Parliament and of the Council of 20 December 2006 on driving licenses (Recast), (2006).
10. Angloinfo. Hungarian Driving Licences 2016 [Available from: https://www.angloinfo.com/hungary/how-to/page/hungary-transport-driving-licences-driving-test.]
11. Citizens Information. Categories of motor vehicles and minimum age of drivers 2016 [Available from: http://www.citizensinformation.ie/en/travel_and_recreation/motoring_1/driver_licensing/categories_of_motor_vehicles_and_minimum_age_of_drivers_in_ireland.html.]
12. Cox DR, Oakes D. Analysis of Survival Data. Cox DR, Hinkley DV, editors. London: Chapman and Hall Ltd; 1984.
13. Davis FG, McCarthy BJ, Freels S, Kupelian V, Bondy ML. The conditional probability of survival of patients with primary malignant brain tumors - Surveillance, epidemiology, and end results (SEER) data. Cancer. 1999;85(2):485-91.
14. Lin CL, Lieu AS, Lee KS, Yang YHC, Kuo TH, Hung MH, et al. The conditional probabilities of survival in patients with anaplastic astrocytoma or glioblastoma multiforme. Surgical Neurology. 2003;60(5):402-6.

For peer review only - http://bmjopen.bmj.com/site/about/guidelines.xhtml

15. Manford M, Hart YM, Sander JW, Shorvon SD. The National General Practice Study of Epilepsy. The syndromic classification of the International League Against Epilepsy applied to epilepsy in a general population. Arch Neurol. 1992;49(8):801-8.
16. Royston P, Ambler G, Sauerbrei W. The use of fractional polynomials to model continuous risk variables in epidemiology. Int J Epidemiol. 1999;28(5):964-74.
17. Royston P, Altman DG. Regression Using Fractional Polynomials of Continuous Covariates - Parsimonious Parametric Modeling. Applied Statistics-Journal of the Royal Statistical Society Series C. 1994;43(3):429-67.
18. Royston P, Sauerbrei W. Multivariable Model-Building - A pragmatic approach to regression analysis based on fractional polynomials for modelling continuous variables: Wiley; 2008.
19. Royston P, Sauerbrei W. Building multivariable regression models with continuous covariates in clinical epidemiology--with an emphasis on fractional polynomials. Methods Inf Med. 2005;44(4):561-71.
20. Stone CJ. Comment: Generalized Additive Models. Statistical Science. 1986;1:3.
21. Schoenfeld D. Partial Residuals for the Proportional Hazards Regression-Model. Biometrika. 1982;69(1):239-41.
22. Akaike H. A New Look at the Statistical Model Identification. Automatic Control, IEEE Transactions on. 1974;19(6):716-23.
23. Collett D. Modelling Survival Data in Medical Research. Boca Raton, Fla.: Chapman & Hall/CRC; 2003.
24. Al-Kattan M, Afifi L, Shamloul R, Mostafa E. Assessment of precipitating factors of breakthrough seizures in epileptic patients2015 July 1, 2015. 165-71 p.
25. Kaddumukasa M, Kaddumukasa M, Matovu S, Katabira E. The frequency and precipitating factors for breakthrough seizures among patients with epilepsy in Uganda. BMC Neurology. 2013;13(1):1-7.
26. Bonnett LJ, Smith CT, Donegan S, Marson AG. Treatment outcome after failure of a first antiepileptic drug. Neurology. 2014;83(6):552-60.
27. Altman DG, Lausen B, Sauerbrei W, Schumacher M. Dangers of using "optimal" cutpoints in the evaluation of prognostic factors. J Natl Cancer Inst. 1994;86(11):829-35.
28. Salinsky M, Wegener K, Sinnema F. Epilepsy, driving laws, and patient disclosure to physicians. Epilepsia. 1992;33(3):469-72.
29. Tudur Smith C, Marson AG, Chadwick DW, Williamson PR. Multiple treatment comparisons in epilepsy monotherapy trials. Trials. 2007;8:34.

FIGURE LEGENDS

Figure 1: Trial Profile

Figure 2: Kaplan-Meier curve for time to next seizure following a breakthrough seizure

ACKNOWLEDGEMENTS & FUNDING

This report is independent research arising from a Post-Doctoral Fellowship (Dr Laura Bonnett - PDF-2015-08-044) supported by the National Institute for Health Research. The views expressed in this publication are those of the authors and not necessarily those of the NHS, the National Institute for Health Research, or the Department of Health.

COMPETING INTERESTS

All authors have completed the ICMJE uniform disclosure form at www.icmje.org/coi_disclosure.pdf and have no interests to declare.

DETAILS OF CONTRIBUTORS

LJB undertook all analyses presented in this manuscript. AGM, CTS and LJB developed the analysis plan and interpreted the analysis results. GP extracted required additional information from the SANAD patient case report forms. All authors drafted and redrafted the manuscript. AGM is the guarantor for this work.

ETHICAL APPROVAL

This was a re-analysis of randomised controlled trial data not requiring ethical approval.

For peer review only - http://bmjopen.bmj.com/site/about/guidelines.xhtml

EXCLUSIVE LICENSE

The Corresponding Author has the right to grant on behalf of all authors and does grant on behalf of all authors, a worldwide licence (http://www.bmj.com/sites/default/files/BMJ%20Author%20Licence%20March%202013.doc) to the Publishers and its licensees in perpetuity, in all forms, formats and media (whether known now or created in the future), to i) publish, reproduce, distribute, display and store the Contribution, ii) translate the Contribution into other languages, create adaptations, reprints, include within collections and create summaries, extracts and/or, abstracts of the Contribution and convert or allow conversion into any format including without limitation audio, iii) create any other derivative work(s) based in whole or part on the on the Contribution, iv) to exploit all subsidiary rights to exploit all subsidiary rights that currently exist or as may exist in the future in the Contribution, v) the inclusion of electronic links from the Contribution to third party material where-ever it may be located; and, vi) licence any third party to do any or all of the above. All research articles will be made available on an Open Access basis (with authors being asked to pay an open access fee—see http://www.bmj.com/about-bmj/resources-authors/forms-policies-and-checklists/copyright-open-access-and-permission-reuse). The terms of such Open Access shall be governed by a Creative Commons licence—details as to which Creative Commons licence will apply to the research article are set out in our worldwide licence referred to above.

DATA SHARING

The anonymised individual participant data from the SANAD study will be made available for research purposes by contacting AGM. Statistical code is available on request from the corresponding author. Participant consent was not required as the presented data are anonymised and risk of identification is low.

For peer review only

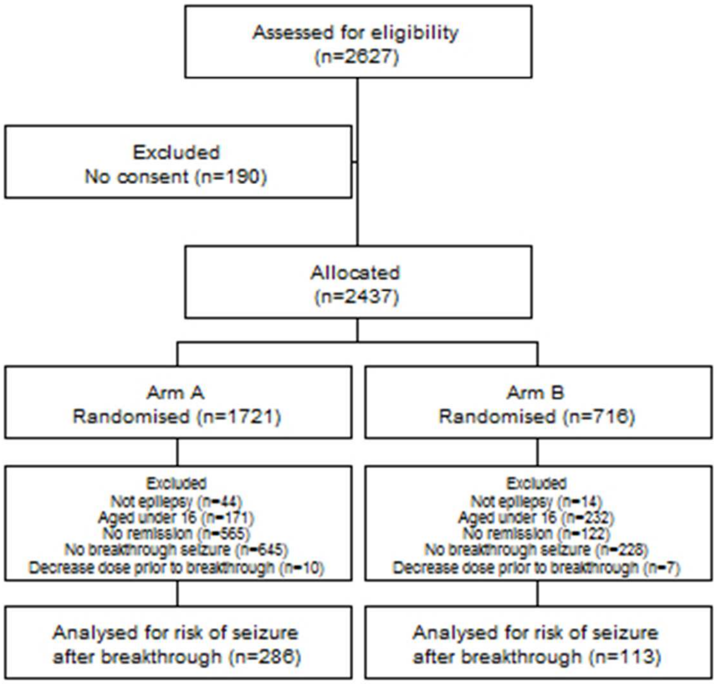

Trial Profile
Figure 1
40x40mm (300 x 300 DPI)

For peer review only

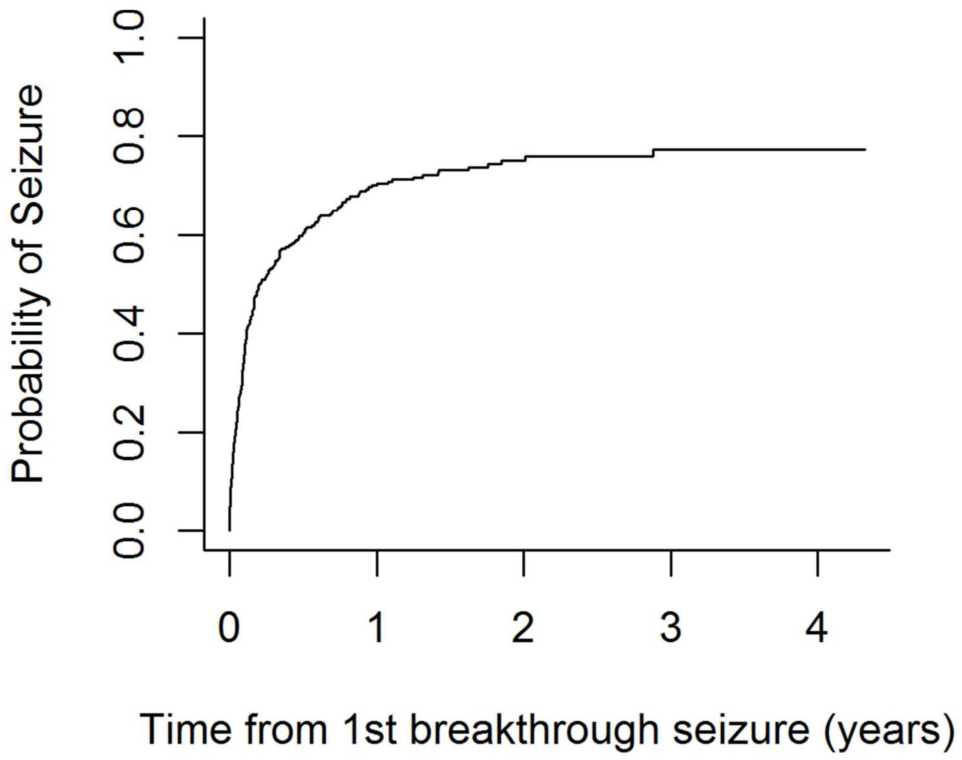

Kaplan-Meier curve for time to next seizure following a breakthrough seizure
Figure 2
99x99mm (300 x 300 DPI)

BMJ Open

Risk of a seizure recurrence after a breakthrough seizure and the implications for driving - further analysis of the Standard versus New Antiepileptic Drugs (SANAD) Randomised Controlled Trial

Journal:	*BMJ Open*
Manuscript ID	bmjopen-2017-015868.R1
Article Type:	Research
Date Submitted by the Author:	31-Mar-2017
Complete List of Authors:	Bonnett, L; University of Liverpool, Biostatistics<br>Powell, Graham; University of Liverpool, Molecular and Clinical Pharmacology<br>Tudur-Smith, Catrin; University of Liverpool, Biostatistics<br>Marson, Anthony; University of Liverpool, Department of Molecular and Clinical Pharmacology
**Primary Subject Heading**:	Neurology
Secondary Subject Heading:	Evidence based practice
Keywords:	Epilepsy < NEUROLOGY, Adult neurology < NEUROLOGY, STATISTICS & RESEARCH METHODS

SCHOLARONE™
Manuscripts

For peer review only

RISK OF A SEIZURE RECURRENCE AFTER A BREAKTHROUGH SEIZURE AND THE IMPLICATIONS FOR DRIVING - FURTHER ANALYSIS OF THE STANDARD VERSUS NEW ANTIEPILEPTIC DRUGS (SANAD) RANDOMISED CONTROLLED TRIAL

LJ BONNETT, GA POWELL, C TUDUR SMITH, AG MARSON

Corresponding Author
Laura J. Bonnett, NIHR Post-Doctoral Fellow
Department of Biostatistics,
Waterhouse Building, Block F,
1-5 Brownlow Street,
Liverpool,
L69 3GL
Email: L.J.Bonnett@liverpool.ac.uk
Telephone: 0151 795 9686

Co-authors
Graham A Powell, MRC Clinical Training Fellow, Department of Molecular and Clinical Pharmacology, Clinical Sciences Centre, Lower Lane, Fazakerley, Liverpool, L9 7LJ. U.K.

Catrin Tudur Smith, Reader in Biostatistics, Department of Biostatistics, Waterhouse Building, Block F, 1-5 Brownlow Street, University of Liverpool, Liverpool, L69 3GL, U.K.

Anthony G Marson, Professor of Neurology, Department of Molecular and Clinical Pharmacology, Clinical Sciences Centre, Lower Lane, Fazakerley, Liverpool, L9 7LJ. U.K.

Key words: Epilepsy, Adults, Driving, Breakthrough Seizures

Word count: 3508

Number of tables: 4

Number of figures: 2

Number of references: 29

ABSTRACT

Objectives: A breakthrough seizure is one occurring after at least 12 months seizure freedom whilst on treatment. The Driver and Vehicle Licensing Agency (DVLA) allow an individual to return to driving once they have been seizure free for 12 months following a breakthrough seizure. This is based on the assumption that the risk of a further seizure in the next 12 months has dropped below 20%. This analysis considers whether the prescribed one year off driving following a breakthrough seizure is sufficient for this, and stratifies risk according to clinical characteristics.

Design, Setting, Participants, Interventions & Main outcome measures: The multi-centre United Kingdom based Standard Versus New Antiepileptic Drug (SANAD) Study was a randomised controlled trial assessing standard and new antiepileptic drugs for patients with newly diagnosed epilepsy. For participants aged at least 16 with a breakthrough seizure, data have been analysed to estimate the annual seizure recurrence risk following a period of six, nine and 12 months seizure freedom. Regression modelling was used to investigate how antiepileptic drug treatment and a number of clinical factors influence the risk of seizure recurrence.

Results: At 12 months following a breakthrough seizure the overall unadjusted risk of a recurrence over the next 12 months is lower than 20%, risk 17% (95% confidence interval: 15% to 19%). However, some patient subgroups have been identified which have an annual recurrence risk significantly greater than 20% after an initial 12 month seizure free period following a breakthrough seizure.

For peer review only - http://bmjopen.bmj.com/site/about/guidelines.xhtml

Conclusions: This reanalysis of SANAD provides estimates of seizure recurrence risks following a breakthrough seizure that will inform policy and guidance about regaining an ordinary driving license. Further guidance is needed as to how such data should be utilised.

Trial Registration: SANAD is registered with the International Standard Randomized Controlled Trial Number Register - ISRCTN38354748.

ARTICLE SUMMARY

Strengths and limitations of this study

- This reanalysis of SANAD provides estimates of seizure recurrence risks following a breakthrough seizure that will inform policy and guidance about regaining an ordinary driving license.
- The SANAD data largely reflects patients with newly diagnosed epilepsy so we have been unable to explore longer term patterns of seizures.
- Patients with epilepsy may elect not to report breakthrough seizures to their clinicians or the relevant driving authority which may lead to an under-estimation of risk.

For peer review only

For peer review only - http://bmjopen.bmj.com/site/about/guidelines.xhtml

INTRODUCTION

A breakthrough seizure is defined as the first seizure after a minimum of 12 months seizure freedom whilst on treatment. The legislation(1) that directs the decisions of the United Kingdom Driver and Vehicle Licensing Agency (DVLA) is informed by a risk based approach. This is summarised in guidance available on their website.(2) In The Motor Vehicle Regulation, epilepsy is defined as a history of two or more clinically unprovoked seizures.(1) According to this, people who have had a breakthrough seizure are usually allowed to regain their group one (ordinary) driving licence one year after the breakthrough seizure provided they have been seizure free, based on the assumption that their risk of a seizure in the next 12 months has fallen below 20%. This minimum level of risk is supported by other European Union member states(3) and has been adopted in the criteria determining minimum driving standards that are being harmonised across the European Union. In the United States each individual state has its own legislation for driving with epilepsy and seizures. When surveyed in 2001(4) most states (n=28) required people with epilepsy to have a time off driving (median six months, range three to 12 months), whereas in 19 states the time was decided by the treating doctor or a medical advisory board.

There are currently few published studies in which seizure recurrence risks are estimated and factors that modify risk investigated. Existing publications(5-8) have focussed on recurrence immediately following a first seizure, or recurrence after treatment withdrawal. Only Bonnett 2010(7) and Bonnett 2011(8) have presented risks of recurrence in the next 12 months following seizure freedom at time points such as six or 12 months. At six months following a first seizure, the risk of another seizure in the next 12 months was 14% (10% to 18%) for those who start antiepileptic drug treatment, and 18% (13% to 23%) for those who do not.(7) At three months after withdrawal of antiepileptic drug treatment following at least 12 months remission from seizures, the risk of a seizure was 15% (10% to 19%).(8) There are no publications considering risk of recurrence following breakthrough seizures. There is therefore a need for reliable published data to inform decisions made by clinicians, DVLA guidance and/or European Union legislation, and legislation outside the European Union.

The Standard versus New Antiepileptic Drugs (SANAD) trial compared standard and new antiepileptic drugs as monotherapy. Arm A recruited 1721 patients who were randomised to treatment with carbamazepine, gabapentin, lamotrigine, topiramate, or oxcarbazepine. Arm B recruited 716 patients who were randomised to lamotrigine, topiramate, or valproate. Patients were followed up to the end of the study whether they remained on their randomised treatment or not, according to the intention to treat principle. Outcomes assessed included time to 12 month remission, time to treatment failure, and time to first seizure.

Here, data from a subset of participants achieving 12 month remission whilst on treatment followed by a breakthrough seizure have been analysed to estimate the subsequent risk of seizure recurrence. Modelling has been used to investigate how a number of clinical factors influence the outcome.

METHODS

Patients

The methods for the SANAD study have been published elsewhere.(9, 10) In summary, patients were eligible for inclusion into SANAD if, in the previous year, they had a history of at least two clinically definite unprovoked epileptic seizures and they were at least five years old. Patients were recruited into Arm A if the recruiting clinician considered carbamazepine to be the optimal standard treatment option. Between December 1st 1999 and June 1st 2001 patients were allocated in a ratio of 1:1:1:1 to carbamazepine, gabapentin, lamotrigine, and topiramate. From 1st June 2001 to 31st August 2004 an oxcarbazepine group was added to the trial and patients were randomly allocated in a ratio of 1:1:1:1:1 to carbamazepine, gabapentin, lamotrigine, oxcarbazepine or topiramate.

Patients were eligible for inclusion in Arm B if the recruiting clinician regarded valproate the standard treatment option. Participants were randomly allocated in a 1:1:1 ratio to valproate, lamotrigine or topiramate between January 12th 1999 and August 31st 2004. The two primary outcomes in SANAD were time to treatment failure from randomisation and time to the first period of 12 months of remission from seizures following randomisation.

For peer review only - http://bmjopen.bmj.com/site/about/guidelines.xhtml

In this paper the Arm A and Arm B datasets have been combined in order to undertake prognostic modelling stratifying by arm. In the original publications trial arms were analysed and reported separately, as the primary purpose was to compare the effectiveness of new antiepileptic drugs with the standard treatments. Here the purpose is different, the aim being to assess the risk of a seizure recurrence following a breakthrough seizure, irrespective of the specific drug that the patient was on at randomisation, or the subsequent choice of treatment.

In order to make the analysis reported here relevant to those of driving age, only participants who achieved 12 month remission whilst on treatment and then had a breakthrough seizure, and were aged 16 years or over when the breakthrough seizures occurred were included. Sixteen years of age was chosen as the lower cut off as by the age 17, after 12 months of follow-up, they would be eligible for a provisional group one license in the United Kingdom. Other European Union countries have a minimum driving age of 18 years(11) with some exceptions such as Hungary(12) and Southern Ireland(13) where the limit is 17 years. In addition, the analysis only included patients who, in the six months prior to their breakthrough seizure, underwent an increase in dosage, or had no change in dosage. In other words, patients with any decrease in dose either with an intention to withdraw, or not, were excluded, as their seizure was likely to be due to antiepileptic drug withdrawal, which is handled differently in the legislation and analyses informing legislation following antiepileptic drug withdrawal have been published.(8)

Statistical Analysis

The outcome of interest is the probability of a seizure recurrence in the next 12 months given that the participants have been seizure free from the breakthrough seizure to the time point in question. For example, the probability of someone who was seizure free for six months after his or her breakthrough seizure, having a seizure in months seven to 18 was calculated by dividing the probability of having a seizure by 18 months by the probability of having a seizure by six months. Risks of recurrence in the next 12 months for other time points were calculated similarly using the Cox model. Confidence intervals for estimates were calculated utilising a revised version of Greenwood's formula.(14-16) Although SANAD was a randomised trial, in this analysis the outcome was measured from the date of the breakthrough seizure, not the date of randomisation.

Variables associated with a higher risk of seizure recurrence were determined univariably and after adjusting for multiple variables using log-rank tests and Cox proportional hazards modelling methods. A best fitting, parsimonious, multivariable model was produced with variable reduction by Akaike's Information Criterion.(17) The recurrence risk in the next 12 months for combinations of risk factors was calculated from the multivariable model.(18) All analyses were undertaken using R 3.2.3.

Continuous variables were investigated using log and fractional polynomial transformations.(19-22) The results for the continuous variables are presented as post-hoc defined categorical variables with categories chosen according to knot positions for a spline model fit to the data.(23) Schoenfeld residual plots(24) and incorporation of time-dependent covariate effects were used to investigate the proportional hazards assumption. The predictive accuracy of the models was assessed using the c-statistic.(25)

Our list of potential prognostic factors included: gender, febrile seizure history, first degree relative with epilepsy, neurological insult, seizure type, epilepsy type, electroencephalogram (EEG) result, computerised tomography (CT) or magnetic resonance imaging (MRI) result, total number of tonic-clonic seizures recorded prior to breakthrough seizure, age at breakthrough seizure, number of treatments required to achieve 12 month remission prior to breakthrough seizure (either monotherapy or polytherapy), time to achieve 12 month remission prior to breakthrough seizure, and breakthrough seizure treatment decision (no change to treatment plan, increase dosage, or decrease dosage for any reason). The breakthrough seizure treatment decision is defined to have occurred up to three months after the seizure and is used as a proxy for the decision that was made at the time of first clinic visit following the breakthrough seizure.

Patients were classified as having neurological insult if they had learning disabilities or neurological deficit, while EEG was classified as normal, not clinically indicated, non-specific abnormality or epileptiform abnormality (focal or generalized spikes or spike and slow wave activity). Seizure types were classified according to the International League Against Epilepsy seizure classification.(26) Epilepsy type was first

For peer review only - http://bmjopen.bmj.com/site/about/guidelines.xhtml

classified as focal, generalised, or unclassified with the unclassified category being used when there was uncertainty between focal onset and generalised onset seizures.

RESULTS

Figure 1 illustrates patient disposition of the 2627 patients recruited into both Arm A and Arm B of SANAD, and identifies patients relevant to this analysis – for the purposes of this analysis, data from both trial arms have been combined. Table 1 summarises the patient demographics for the 399 patients under analysis. Of these patients, 254 experienced at least one further seizure after breakthrough. Patients in Arm A were followed up for a median of 1.67 years following a breakthrough seizure (interquartile range (IQR) 0.85 to 2.59 years) while patients in Arm B were followed up for a median of 1.41 years (IQR 0.55 to 2.56 years). In total there were 705.6 patient years of follow-up after the breakthrough seizure.

Figure 2 illustrates the risk of seizure recurrence after a breakthrough seizure. The median time to a further seizure following a breakthrough was 76 days (IQR 57 to 122 days). The probability of a seizure by 12 months was 70.1%. In particular, 111 (28%) people had had a seizure by one month, 166 (42%) by two months, 214 (54%) by six months, 242 (61%) by one year, 252 (63%) by two years and 254 (64%) by the end of the follow-up period. Table 2 shows unadjusted 12 month seizure recurrence risks at various time points after the breakthrough seizure. At six months the estimate is significantly above 20%. At 12 months however, the estimate is below 20% and significantly so as the 95% confidence interval does not include 20%.

Table 1: Patient demographics

Characteristic (n (%) unless otherwise stated)	Arm A (n=286)	Arm B (n=113)	Total (n=399)
Male	159 (56)	72 (64)	231 (58)
Febrile seizure history	15 (5)	5 (4)	20 (5)
Epilepsy in first degree relative	24 (8)	21 (19)	45 (11)
Neurological insult	38 (13)	9 (8)	47 (12)
Seizures			
Simple or complex partial with secondary generalised seizures	180 (63)	5 (4)	185 (46)
Simple or complex partial only	72 (25)	1 (0)	73 (18)
Generalised tonic-clonic seizures only	4 (1)	32 (29)	36 (9)
Absence seizures	1 (0)	5 (5)	6 (2)
Myoclonic or absence seizures with tonic- clonic seizures	0 (0)	28 (25)	28 (7)
Tonic-clonic seizures, uncertain if focal or generalised	27 (10)	34 (30)	61 (15)
Other	2 (1)	8 (7)	10 (3)
Epilepsy type			
Partial	253 (88)	6 (5)	259 (65)
Generalised	6 (2)	69 (61)	75 (19)
Unclassified	27 (10)	38 (34)	65 (16)
EEG results			
Normal	134 (47)	32 (28)	166 (42)
Non-specific abnormality	49 (17)	13 (12)	62 (16)
Epileptiform abnormality	69 (24)	64 (57)	133 (33)
Not clinically indicated	34 (12)	4 (3)	38 (9)
CT/MRI scan results			
Normal	164 (57)	59 (52)	223 (56)
Abnormal	75 (26)	10 (9)	85 (21)
Not clinically indicated	47 (17)	44 (39)	91 (23)
Number of treatments required to achieve 12 month remission			
Monotherapy	219 (77)	86 (77)	305 (77)
Polytherapy	67 (23)	27 (23)	94 (23)
Number of tonic-clonic seizures reported by first breakthrough seizure, median (IQR)	3 (1, 6)	3 (2, 6)	3 (1, 6)
Age at first breakthrough seizure, median (IQR)	44.5 (31.8, 57.7)	24.0 (21.1, 34.5)	38.3 (24.5, 53.5)
Time to achieve 12 month remission prior to breakthrough seizure (years), median (IQR)	1.2 (1.0, 1.9)	1.1 (1.0, 1.8)	1.2 (1.0, 1.9)
Treatment decision prior to breakthrough seizure			
No change to treatment plan	261 (91)	101 (89)	362 (91)
Increase dosage	25 (9)	12 (11)	37 (9)

For peer review only - http://bmjopen.bmj.com/site/about/guidelines.xhtml

Breakthrough seizure treatment decision			
No change to treatment plan	169 (61)	67 (61)	236 (61)
Increase dosage	99 (36)	40 (37)	139 (36)
Decrease dosage for any reason, or missing decision	9 (3)	2 (2)	11 (3)

Table 2: Unadjusted 12 month seizure recurrence risks at time points after breakthrough seizure: risk (%, 95% Confidence Interval)

Time seizure free after breakthrough seizure (months)	Number at Risk	Risk of seizure in following 12 months. %
6	119	32 (28 to 36)
9	99	24 (21 to 27)
12	80	17 (15 to 19)

Results for univariable and multivariable modelling of time to seizure recurrence are presented in Table 3. In the univariable model, number of drugs required to achieve initial 12 month remission and time to achieve a first 12 month remission prior to breakthrough seizure were associated with seizure recurrence risk – patients requiring polytherapy to achieve remission were more likely to have a recurrence than those requiring monotherapy. Additionally, patients achieving remission immediately at one year were less likely to have a recurrence following a breakthrough seizure than those who took longer to achieve 12 month remission. Breakthrough seizure treatment decision was also associated with the outcome; patients having an increase in dose after their breakthrough seizure were more likely to have a recurrence than those not changing their treatment, which may be counter intuitive, but indicates clinicians are able to identify those at higher recurrence risk.

The final multivariable model included number of drugs required to achieve initial remission, time to achieve initial 12 month remission and breakthrough seizure treatment decision. As before – patients requiring polytherapy to achieve remission were more likely to have a recurrence than those requiring monotherapy, patients achieving remission immediately at one year were less likely to have a recurrence than those who took longer to achieve 12 month remission, and patients increasing their dose after their breakthrough seizure were more likely to have a recurrence than those not changing their dose. There was no evidence to suggest that the proportional hazards assumption, underlying the Cox model, was invalid. The c-statistic for the model was 0.62, indicating that the model accurately discriminates participants 62% of the time, which is reasonable internal validation.(27, 28)

Breakthrough seizure treatment decision, although significantly associated with the outcome, should not be considered as a modifiable variable, as clinicians will find it very difficult to use this information to inform treatment decisions for future patients. Therefore the model was refitted excluding this covariate, and the resulting parsimonious model included number of drugs attempted to achieve initial 12 month remission, and time taken to achieve initial 12 month remission. The direction of the effects remained unchanged (Table 3).

The risk of recurrence at 12 months for patients with particular characteristics was estimated from the parsimonious multivariable regression model. Results can be seen in Table 4. At six months seizure freedom following a breakthrough seizure no patient subgroups had a risk of recurrence that was below 20%. By 12 months of seizure freedom, the current recommended time off driving following a breakthrough seizure, several patient subgroups still had estimates in excess of the 20%. In particular, the length of time required for the estimate of seizure recurrence to fall below 20% for patients requiring polytherapy to achieve initial 12 month remission, and taking three or more years to enter initial period of 12 month remission is 15 months.

Table 3: Effect estimates from univariable and multivariable models [TC = tonic-clonic; Gen = generalised]

Variable	Comparison	Univariable p-value	Univariable HR (95% CI)	Multivariable HR (95% CI)	Multivariable HR (95% CI) w/o decision variable
Gender	Female<br>Male	0.43	1.00<br>1.11 (0.86, 1.42)	N/A	N/A

For peer review only - http://bmjopen.bmj.com/site/about/guidelines.xhtml

Febrile seizure history	Absent	0.28	1.00	N/A	N/A
	Present		0.69 (0.35, 1.34)		
Epilepsy in 1st degree relative	Absent	0.82	1.00	N/A	N/A
	Present		1.05 (0.69, 1.59)		
Neurological insult	Absent	0.59	1.00	N/A	N/A
	Present		0.90 (0.62, 1.32)		
Seizure type	Simple/complex partial + 2° gen.		1.00	N/A	N/A
	Simple/complex partial only	0.35	1.17 (0.84, 1.63)		
	Generalised TC only	0.65	0.87 (0.48, 1.58)		
	Absence	0.96	1.03 (0.35, 3.03)		
	Myoclonic/absence + TC	1.00	1.00 (0.49, 2.03)		
	TC (uncertain if focal or gen.)	0.49	0.85 (0.54, 1.34)		
	Other	0.89	1.07 (0.44, 2.55)		
Epilepsy type	Partial		1.00	N/A	N/A
	Generalised	0.65	0.88 (0.52, 1.49)		
	Unclassified	0.55	0.88 (0.57, 1.35)		
EEG results	Normal		1.00	N/A	N/A
	Non-specific Abnormality	0.62	0.91 (0.63, 1.32)		
	Epileptiform Abnormality	0.87	0.98 (0.73, 1.30)		
	Not done/Missing	0.05	0.60 (0.36, 1.00)		
CT/MRI scan results	Normal		1.00	N/A	N/A
	Abnormal	0.15	0.79 (0.57, 1.09)		
	Not done/Missing	0.86	0.97 (0.71, 1.33)		
No. drugs attempted for remission	Monotherapy	0.01	1.00	1.00	1.00
	Polytherapy		1.47 (1.11, 1.94)	1.37 (1.02, 1.84)	1.28 (0.96, 1.71)
Number of tonic-clonic seizures reported by first breakthrough seizure [Linear]	0	0.60	1.00	N/A	N/A
	1		1.00 (1.00, 1.00)		
	2		1.00 (1.00, 1.01)		
	3-4		1.00 (0.99, 1.01)		
	5-6		1.00 (0.99, 1.02)		
	7-10		1.01 (0.98, 1.03)		
	11-20		1.01 (0.97, 1.05)		
	>20		1.31 (0.48, 3.52)		
Age at first breakthrough seizure [Linear]	≤ 20	0.39	1.00	N/A	N/A
	21-30		1.02 (0.97, 1.07)		
	31-45		1.07 (0.92, 1.23)		
	46-70		1.14 (0.85, 1.53)		
	> 70		1.22 (0.78, 1.89)		
Time to achieve initial 12 month remission (years) [FP]	1	<0.001	1.00	1.00	1.00
	1-1.5		1.27 (1.12, 1.44)	1.21 (1.06, 1.38)	1.24 (1.08, 1.41)
	1.5-2		1.57 (1.24, 1.98)	1.43 (1.12, 1.82)	1.49 (1.16, 1.89)
	2-3		1.75 (1.31, 2.34)	1.56 (1.15, 2.11)	1.64 (1.21, 2.22)
	>3		1.89 (1.36, 2.62)	1.65 (1.17, 2.43)	1.75 (1.24, 2.46)
Breakthrough seizure decision	No change to treatment plan		1.00	1.00	N/A
	Increase dosage	<0.001	2.05 (1.59, 2.66)	2.05 (1.59, 2.66)	
	Decrease dosage (or not specified)	0.83	1.07 (0.59, 1.93)	0.99 (0.55, 1.79)	

HR>1 – seizure recurrence more likely; [FP] implies fractional polynomial transformation of this covariate; [Linear] implies no transformation of this covariate

Table 4: Risk of seizure recurrence in next 12 months estimated from multivariable model at specific seizure-free periods.

Patient Characteristics		Duration of seizure freedom after breakthrough seizure (months)	Risk of seizure in next 12 months (%, 95% CI)	Months of seizure freedom required from breakthrough seizure until annual risk falls <20%
No. drugs required to achieve remission prior to breakthrough seizure	Time to achieve 12 month remission (years) prior to breakthrough seizure			
Monotherapy	1	6	20 (10 to 31)	6.1

For peer review only - http://bmjopen.bmj.com/site/about/guidelines.xhtml

		9<br>12<br>18	15 (4 to 25)<br>10 (0 to 21)<br>6 (0 to 16)	
Monotherapy	2	6<br>9<br>12<br>18	30 (21 to 39)<br>22 (13 to 32)<br>16 (6 to 26)<br>10 (0 to 19)	10.6
Monotherapy	3	6<br>9<br>12<br>18	32 (23 to 41)<br>24 (15 to 33)<br>17 (8 to 27)<br>11 (1 to 20)	11.1
Monotherapy	4	6<br>9<br>12<br>18	33 (24 to 42)<br>25 (16 to 34)<br>18 (8 to 27)<br>11 (1 to 20)	11.1
Polytherapy	2	6<br>9<br>12<br>18	37 (29 to 45)<br>28 (19 to 30)<br>20 (11 to 30)<br>12 (3 to 22)	13.2
Polytherapy	3	6<br>9<br>12<br>18	40 (32 to 48)<br>30 (22 to 39)<br>22 (13 to 31)<br>13 (4 to 23)	15.0
Polytherapy	4	6<br>9<br>12<br>18	41 (33 to 48)<br>31 (22 to 39)<br>22 (13 to 31)<br>14 (5 to 23)	15.8

For peer review only

DISCUSSION

In the United Kingdom the DVLA prescribe one year off driving following a breakthrough seizure based on legislation and the assumption that a person's risk of a seizure in the next 12 months is below 20%. According to data from the SANAD study, the overall risk of a seizure recurrence, unadjusted for any covariates, falls significantly below 20% by 12 months of seizure freedom following the breakthrough seizure as required. Covariates significantly associated with the outcome were time taken to achieve an initial 12 month remission, number of drugs required to achieve that remission, and breakthrough seizure treatment decision. As expected, those patients who achieve a period of 12 month remission quickly, and those patients who require only one drug to achieve remission, had a lower chance of a seizure recurrence.

The decision to not change antiepileptic drug dose following a breakthrough seizure was associated with a lower risk of a recurrence than the decision to increase dosage. This result is potentially counter-intuitive as one might expect an increase in dose to reduce seizure risk. However, it is likely that clinicians are able to identify patients at higher risk of recurrence and recommend treatment changes to reduce that risk, although additional relevant clinical factors have not been identified by our model, and this requires further investigation. It is important to highlight that in most cases, the decision to increase dose was taken in between neurology clinic appointments at which follow-up data were collected, presumably at the advice of the GP or neurologist. As a result, accurate dates of dose increase have not been recorded and it is possible that a subgroup of patients had further seizures following the initial breakthrough seizure, prompting the clinician to increase the antiepileptic drug dose. When breakthrough seizure treatment decision was removed from the list of candidate variables to reflect the fact that clinicians will find it very difficult to use this information to inform treatment decisions for future patients, the parsimonious model included covariates for number of drugs required to achieve an initial 12 month remission, and time taken to achieve initial 12 month remission. Only patients requiring polytherapy to achieve initial 12 month remission and taking at least two years to achieve initial 12 month remission required longer than 12 months for their risk of a subsequent seizure to be less than 20%. This suggests that the current 12 month time off driving is generally appropriate. Even in the high risk groups, the recurrence risks are fairly close to 20% if the focus is on point estimates.

Few publications have considered risk of a breakthrough seizure and tend to be focused on patients in developing countries.(29, 30) A study of 256 patients in Uganda identified non-compliance to antiepileptic drug

For peer review only - http://bmjopen.bmj.com/site/about/guidelines.xhtml

therapy, duration of treatment, infections, and menses among female study participants as factors significantly associated with breakthrough seizures.(30) Precipitating factors for breakthrough seizures for a study of 90 patients in Egypt were missed doses, sleep deprivation and psychological stress, although the authors also found differences in duration of seizure control, number of antiepileptic drugs and abnormal epileptic activity in EEG between patients with and without breakthrough seizures.(29) These factors were not collected as part of the SANAD study and as such have not been considered as part of this analysis. Neither study considered outcomes following the breakthrough study. We are unaware of any studies looking at outcome after a breakthrough seizure. In particular, we have been unable to identify any prognostic models considering risk of seizure recurrence following a breakthrough seizure for patients of driving age in developed countries. Another analysis of SANAD for patients of driving age has considered risk of a second treatment failure after a first.(31)

Others who have investigated driving regulations for patients with epilepsy have considered the time off driving required until the risk of seizure recurrence falls below 2.5% per month.(32) This corresponds to a monthly risk of a seizure while driving of 1.04 per thousand and equates to eight months off driving following an unprovoked first-ever seizure. Although the outcome under consideration in our manuscript is breakthrough seizure after remission rather than first ever seizure, the time off driving is fairly consistent across the papers.

Limitations

SANAD recruited a large number of patients and followed them up for a long period – up to six years in some cases. However, only a small subset of these patients was relevant to address the question of risk of a seizure recurrence following a breakthrough seizure for patients of driving age. The requirement of patients to achieve initial remission of at least 12 months and then have a breakthrough seizure to be included in this analysis also meant that the follow-up of patients after the breakthrough seizure was relatively short. This means that some confidence intervals associated with the risk estimates are quite wide. Additionally, the SANAD data largely reflects patients with newly diagnosed epilepsy. We have therefore been unable to explore longer term patterns. For example, if patients go into and out of remission then their seizure recurrence risks might change compared to these estimates. The subset of patients considered for this analysis may also have limited power to detect some prognostic effects as significant. Other important factors may exist which have not been analysed, or collected. The SANAD study also indicated that lamotrigine was superior to carbamazepine in terms of seizure control for partial onset seizures.(10) Given the relatively small sample size, we have had to combine treatment groups for our analysis rather than undertake per-drug analyses and thus assume that combining groups is clinically valid.

The multivariable model for risk of seizure recurrence included a continuous covariate – time to achieve initial 12 month remission. Therefore, to estimate the risk of recurrence over the next 12 months for combinations of risk factors including this covariate, the variable had to be categorised which may not be the most efficient approach.(33) Also, neurological insult, seizure type, epilepsy type, and CT/MRI scan result were recorded at baseline rather than at the breakthrough seizure. Although these covariates may have changed by a breakthrough seizure, it is likely that any change occurred in only a small number of patients. EEG was also only recorded at baseline, and it is possible that EEG on treatment would be prognostic, although given the unpredictable nature of breakthrough seizures, it would not be feasible to undertake an EEG in order to inform risk.

There is evidence to suggest that patients with epilepsy may elect not to report breakthrough seizures to their clinicians or the relevant driving authority.(34) The evidence collected as part of SANAD is patient reported seizure counts and therefore our results may be under-estimating the actual risk. Increased patient counselling regarding the risks involved with driving, the need for driving regulations, and the importance of compliance with these rules may only have a limited impact as the implications for patients losing their driving license are potentially serious such as job losses, and resulting lack of independence. The model developed here should ideally be validated in other similar datasets. However, no other similar datasets exist. The best match is a set of individual participant data we have collected.(35) These data include only very small numbers of relevant patients. Therefore, alternative data sources are required.

Conclusions

Twelve months appears to be an appropriate time off driving for patients of driving age who have experienced a period of at least 12 months initial seizure freedom followed by a breakthrough seizure. Provided that patients

For peer review only - http://bmjopen.bmj.com/site/about/guidelines.xhtml

remain seizure free for 12 months following a breakthrough seizure, their risk of a seizure in the next 12 months would be less than the 20% risk standard that informs the UK legislation and DLVA guidance.

As discussed in depth in Bonnett 2010(7), the legislators and DVLA need to decide whether to base time off driving on unadjusted estimates only, or whether they should consider estimates adjusted for important clinical factors. Although our unadjusted results suggest that 12 months off driving is sufficient time off driving, risk estimates differ substantially among groups. For some patient subgroups at least 15 months off driving is required for their point estimate to reduce below 20%. Additionally, discussions are required to determine whether associated 95% confidence intervals should be used to inform the decision making process. The unadjusted risk estimate is significantly below 20% by 12 months. However, none of the adjusted risk estimates are significantly below 20% by 12 months.

Evidence is inconclusive regarding whether drivers with epilepsy have higher rates of motor vehicle accidents than those without epilepsy. However there is evidence that accidents are 26 times more likely to occur with drivers with other medical conditions compared to drivers with epilepsy.(36) Implementing a policy based on clinical factors is potentially challenging. In fact, in practice time to achieve remission may be the only factor that could be incorporated into such an assessment as there is potential for manipulation of drugs in terms of number and doses to meet driving objectives. Furthermore, introducing a tiered system may compromise patient care as patients would be inclined to 'fit in' to the shorter duration if driving is important to them.

REFERENCES

1. The Motor Vehicles (Driving Licences) (Amendment) Regulations 2013, (2013).
2. Driver and Vehicle Licensing Agency. Assessing fitness to drive - a guide for medical professionals. In: Group DM, editor. 2016.
3. Schmedding E. Epilepsy and Driving in Belgium: Proposals and Justification. Acta Neurologica Belgica. 2004;104(2):68-79.
4. Krauss GL, Ampaw L, Krumholz A. Individual state driving restrictions for people with epilepsy in the US. Neurology. 2001;57(10):1780-5.
5. Kim LG, Johnson TL, Marson AG, Chadwick DW. Prediction of Risk of Seizure Recurrence after a Single Seizure and Early Epilepsy: Further Results from the MESS Trial. The Lancet Neurology. 2006;5(4):317-22.
6. Prognosis of Epilepsy in Newly Referred Patients: A Multicenter Prospective Study of the Effects of Monotherapy on the Long-Term Course of Epilepsy. Collaborative Group for the Study of Epilepsy. Epilepsia. 1992;33(1):45-51.
7. Bonnett LJ, Tudur-Smith C, Williamson PR, Marson AG. Risk of recurrence after a first seizure and implications for driving: further analysis of the Multicentre study of early Epilepsy and Single Seizures. BMJ. 2010;341:c6477.
8. Bonnett LJ, Shukralla A, Tudur-Smith C, Williamson PR, Marson AG. Seizure recurrence after antiepileptic drug withdrawal and the implications for driving: further results from the MRC Antiepileptic Drug Withdrawal Study and a systematic review. Journal of Neurology Neurosurgery and Psychiatry. 2011;82(12):1328-33.
9. Marson AG, Al-Kharusi AM, Alwaidh M, Appleton R, Baker GA, Chadwick DW, et al. The SANAD study of effectiveness of carbamazepine, gabapentin, lamotrigine, oxcarbazepine, or topiramate for treatment of partial epilepsy: an unblinded randomised controlled trial. Lancet. 2007;369(9566):1000-15.
10. Marson AG, Al-Kharusi AM, Alwaidh M, Appleton R, Baker GA, Chadwick DW, et al. The SANAD study of effectiveness of valproate, lamotrigine, or topiramate for generalised and unclassifiable epilepsy: an unblinded randomised controlled trial. Lancet. 2007;369(9566):1016-26.
11. Directive 2006/126/EC of the European Parliament and of the Council of 20 December 2006 on driving licenses (Recast), (2006).
12. Angloinfo. Hungarian Driving Licences 2016 [Available from: https://www.angloinfo.com/hungary/how-to/page/hungary-transport-driving-licences-driving-test].
13. Citizens Information. Categories of motor vehicles and minimum age of drivers 2016 [Available from: http://www.citizensinformation.ie/en/travel_and_recreation/motoring_1/driver_licensing/categories_of_motor_vehicles_and_minimum_age_of_drivers_in_ireland.html].
14. Cox DR, Oakes D. Analysis of Survival Data. Cox DR, Hinkley DV, editors. London: Chapman and Hall Ltg; 1984.

For peer review only - http://bmjopen.bmj.com/site/about/guidelines.xhtml

15. Davis FG, McCarthy BJ, Freels S, Kupelian V, Bondy ML. The conditional probability of survival of patients with primary malignant brain tumors - Surveillance, epidemiology, and end results (SEER) data. Cancer. 1999;85(2):485-91.
16. Lin CL, Lieu AS, Lee KS, Yang YHC, Kuo TH, Hung MH, et al. The conditional probabilities of survival in patients with anaplastic astrocytoma or glioblastoma multiforme. Surgical Neurology. 2003;60(5):402-6.
17. Akaike H. A New Look at the Statistical Model Identification. Automatic Control, IEEE Transactions on. 1974;19(6):716-23.
18. Collett D. Modelling Survival Data in Medical Research. Boca Raton, Fla.: Chapman & Hall/CRC; 2003.
19. Royston P, Ambler G, Sauerbrei W. The use of fractional polynomials to model continuous risk variables in epidemiology. Int J Epidemiol. 1999;28(5):964-74.
20. Royston P, Altman DG. Regression Using Fractional Polynomials of Continuous Covariates - Parsimonious Parametric Modeling. Applied Statistics-Journal of the Royal Statistical Society Series C. 1994;43(3):429-67.
21. Royston P, Sauerbrei W. Multivariable Model-Building - A pragmatic approach to regression analysis based on fractional polynomials for modelling continuous variables: Wiley; 2008.
22. Royston P, Sauerbrei W. Building multivariable regression models with continuous covariates in clinical epidemiology--with an emphasis on fractional polynomials. Methods Inf Med. 2005;44(4):561-71.
23. Stone CJ. Comment: Generalized Additive Models. Statistical Science. 1986;1:3.
24. Schoenfeld D. Partial Residuals for the Proportional Hazards Regression-Model. Biometrika. 1982;69(1):239-41.
25. Harrell FE, Lee KL, Mark DB. Multivariable prognostic models: Issues in developing models, evaluating assumptions and adequacy, and measuring and reducing errors. Statistics in Medicine. 1996;15(4):361-87.
26. Manford M, Hart YM, Sander JW, Shorvon SD. The National General Practice Study of Epilepsy. The syndromic classification of the International League Against Epilepsy applied to epilepsy in a general population. Arch Neurol. 1992;49(8):801-8.
27. Hosmer DW, Lemeshow S. Applied Logistic Regression. Second ed. New York: John Wiley & Sons; 2000.
28. Harrell FE, Lee KL, Califf RM, Pryor DB, Rosati RA. Regression Modeling Strategies for Improved Prognostic Prediction. Statistics in Medicine. 1984;3(2):143-52.
29. Al-Kattan M, Afifi L, Shamloul R, Mostafa E. Assessment of precipitating factors of breakthrough seizures in epileptic patients2015 July 1, 2015. 165-71 p.
30. Kaddumukasa M, Kaddumukasa M, Matovu S, Katabira E. The frequency and precipitating factors for breakthrough seizures among patients with epilepsy in Uganda. BMC Neurology. 2013;13(1):1-7.
31. Bonnett LJ, Smith CT, Donegan S, Marson AG. Treatment outcome after failure of a first antiepileptic drug. Neurology. 2014;83(6):552-60.
32. Brown JW, Lawn ND, Lee J, Dunne JW. When is it safe to return to driving following first-ever seizure? J Neurol Neurosurg Psychiatry. 2015;86(1):60-4.
33. Altman DG, Lausen B, Sauerbrei W, Schumacher M. Dangers of using "optimal" cutpoints in the evaluation of prognostic factors. J Natl Cancer Inst. 1994;86(11):829-35.
34. Salinsky M, Wegener K, Sinnema F. Epilepsy, driving laws, and patient disclosure to physicians. Epilepsia. 1992;33(3):469-72.
35. Tudur Smith C, Marson AG, Chadwick DW, Williamson PR. Multiple treatment comparisons in epilepsy monotherapy trials. Trials. 2007;8:34.
36. Naik PA, Fleming ME, Bhatia P, Harden CL. Do drivers with epilepsy have higher rates of motor vehicle accidents than those without epilepsy? Epilepsy & Behavior.47:111-4.

FIGURE LEGENDS

Figure 1: SANAD Trial Profile

Figure 2: Kaplan-Meier curve for time to next seizure following a breakthrough seizure

ACKNOWLEDGEMENTS & FUNDING

This report is independent research arising from a Post-Doctoral Fellowship (Dr Laura Bonnett - PDF-2015-08-044) supported by the National Institute for Health Research. The views expressed in this publication are those of the authors and not necessarily those of the NHS, the National Institute for Health Research, or the Department of Health.

For peer review only - http://bmjopen.bmj.com/site/about/guidelines.xhtml

COMPETING INTERESTS

All authors have completed the ICMJE uniform disclosure form at www.icmje.org/coi_disclosure.pdf and have no interests to declare.

DETAILS OF CONTRIBUTORS

LJB undertook all analyses presented in this manuscript. AGM, CTS and LJB developed the analysis plan and interpreted the analysis results. GP extracted required additional information from the SANAD patient case report forms. All authors drafted and redrafted the manuscript. AGM is the guarantor for this work.

ETHICAL APPROVAL

This was a re-analysis of randomised controlled trial data not requiring ethical approval.

EXCLUSIVE LICENSE

The Corresponding Author has the right to grant on behalf of all authors and does grant on behalf of all authors, a worldwide licence (http://www.bmj.com/sites/default/files/BMJ%20Author%20Licence%20March%202013.doc) to the Publishers and its licensees in perpetuity, in all forms, formats and media (whether known now or created in the future), to i) publish, reproduce, distribute, display and store the Contribution, ii) translate the Contribution into other languages, create adaptations, reprints, include within collections and create summaries, extracts and/or, abstracts of the Contribution and convert or allow conversion into any format including without limitation audio, iii) create any other derivative work(s) based in whole or part on the on the Contribution, iv) to exploit all subsidiary rights to exploit all subsidiary rights that currently exist or as may exist in the future in the Contribution, v) the inclusion of electronic links from the Contribution to third party material where-ever it may be located; and, vi) licence any third party to do any or all of the above. All research articles will be made available on an Open Access basis (with authors being asked to pay an open access fee—see http://www.bmj.com/about-bmj/resources-authors/forms-policies-and-checklists/copyright-open-access-and-permission-reuse). The terms of such Open Access shall be governed by a Creative Commons licence—details as to which Creative Commons licence will apply to the research article are set out in our worldwide licence referred to above.

DATA SHARING

The anonymised individual participant data from the SANAD study will be made available for research purposes by contacting AGM. Statistical code is available on request from the corresponding author. Participant consent was not required as the presented data are anonymised and risk of identification is low.

For peer review only

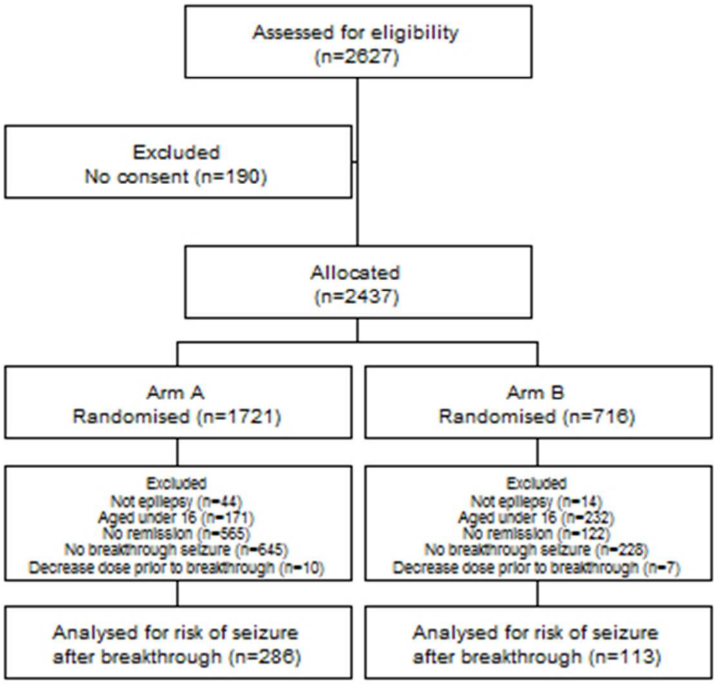

Trial Profile

40x40mm (300 x 300 DPI)

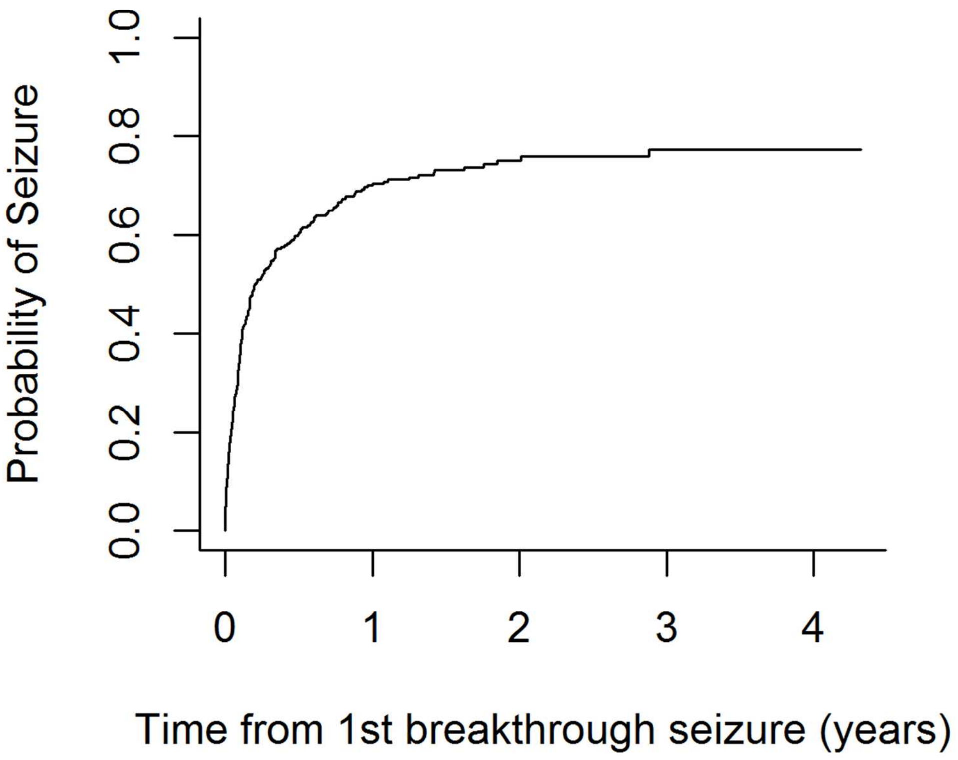

Kaplan-Meier curve for time to next seizure following a breakthrough seizure

99x99mm (300 x 300 DPI)

ly
